# Association between proximity to and coverage of traditional fast-food restaurants and non-traditional fast-food outlets and fast-food consumption among rural adults

**DOI:** 10.1186/1476-072X-10-37

**Published:** 2011-05-20

**Authors:** Joseph R Sharkey, Cassandra M Johnson, Wesley R Dean, Scott A Horel

**Affiliations:** 1 Program for Research in Nutrition and Health Disparities, School of Rural Public Health, Texas A&M Health Science Center, MS 1266, College Station, TX, 77843-1266, USA; 2 Program on GIS and Spatial Statistics, School of Rural Public Health, Texas A&M Health Science Center, MS 1266, College Station, TX, 77843-1266, USA

## Abstract

**Objective:**

The objective of this study is to examine the relationship between residential exposure to fast-food entrées, using two measures of potential spatial access: proximity (distance to the nearest location) and coverage (number of different locations), and weekly consumption of fast-food meals.

**Methods:**

Traditional fast-food restaurants and non-traditional fast-food outlets, such as convenience stores, supermarkets, and grocery stores, from the 2006 Brazos Valley Food Environment Project were linked with individual participants (*n *= 1409) who completed the nutrition module in the 2006 Brazos Valley Community Health Assessment.

**Results:**

Increased age, poverty, increased distance to the nearest fast food, and increased number of different traditional fast-food restaurants, non-traditional fast-food outlets, or fast-food opportunities were associated with less frequent weekly consumption of fast-food meals. The interaction of gender and proximity (distance) or coverage (number) indicated that the association of proximity to or coverage of fast-food locations on fast-food consumption was greater among women and opposite of independent effects.

**Conclusions:**

Results provide impetus for identifying and understanding the complex relationship between access to all fast-food opportunities, rather than to traditional fast-food restaurants alone, and fast-food consumption. The results indicate the importance of further examining the complex interaction of gender and distance in rural areas and particularly in fast-food consumption. Furthermore, this study emphasizes the need for health promotion and policy efforts to consider all sources of fast-food as part of promoting healthful food choices.

## Introduction

Fast food is characterized by low cost, large portion size, and energy-dense foods that are high in calories and total fat [1-5]. Individuals and families seek fast food for many reasons, particularly because of time and budget; fast food is cheap, quick, easy to access, and tasty [6-13]. Emerging research reports that fast-food consumption, more so than general away-from-home food consumption, is associated with undesirable metabolic outcomes, including higher body weight, waist circumference, and insulin resistance [11,14-18]. Some socioeconomically and geographically disadvantaged populations have relatively better access to fast-food outlets and convenience stores than to supermarkets, which may increase exposure to unhealthy foods and negatively influence diet and health [19-24].

Research findings indicate that fast-food outlets dominate the food landscape and that rural residents can readily find fast-food items for consumption [24-26]. In a study in a large rural area of Texas, Creel and colleagues found that 59% of the food locations offering fast-food items were not traditional fast-food restaurants, but convenience stores, supermarkets, and grocery stores [26], which increased spatial access to fast-food items [24]. This illustrates the concept of "channel blurring", where stores expand food items to include new layouts and product lines, including fast food. Recently, many convenience stores and supermarkets/grocery stores incorporated fast food into their food offerings, thereby increasing the variety of shopping venues and food options that are available to their consumers [27-29]. Still, research continues to focus on fast-food restaurants as the sole source of fast food. Specifically, the literature has concentrated its attention on the association between obesity and access to fast-food restaurants, often described as the density of fast-food restaurants, with mixed results [10,11,15,16,19,30-34]. There are a limited number of studies that examined fast-food exposure and consumption, and apparently no studies that examined the relationship between dietary intake and exposure to fast food from all food outlets that market fast food [34,35]. Although no rural studies have established significance for the relationship between access to fast food and fast-food consumption, mounting evidence from the literature suggests that rural areas are obesogenic, characterized by poor local availability of healthy foods, limited access to transportation, and widespread fast-food opportunities [19-22,26,36]. "Ground-truthed" methodology, which includes on-site observation and Global Positioning System (GPS) data, enhances the measurement of access to food resources, especially in rural areas [27]. Because little is known about potential spatial access to fast food within rural areas and its association with frequency of fast-food meal consumption, the objective of this study is to examine the relationship between residential access to fast food, using two measures of potential spatial access - proximity (distance to the nearest location) and coverage (variety or number of different locations) [27], and weekly consumption of fast-food meals among 1,409 men and women who reside in a large rural area of Texas.

## Methods

### Geographic setting

**Figure 1:**
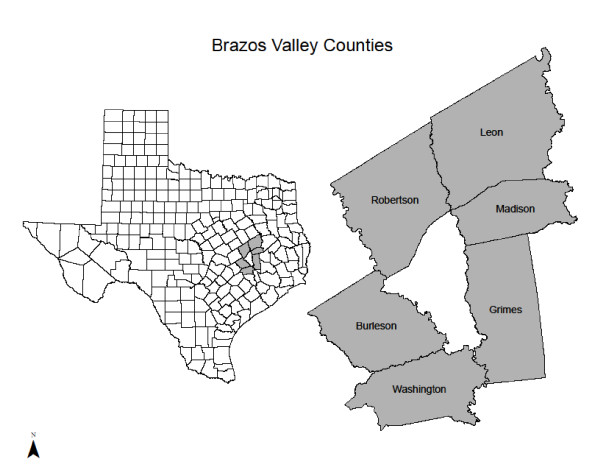
**Map of Brazos Valley Counties**.

The study used data from the 2006 Brazos Valley Food Environment Project (BVFEP) and the 2006 Brazos Valley Community Health Assessment (BVHA), which were approved by the Institutional Review Board at Texas A&M University for six rural counties in the Central Texas Brazos Valley region (see Figure 1). These counties include five urban clusters (population 3,181-11,952), and are considered rural based on population density (persons/mi^2^) [37]. The rural region covers a land area of 4,466 mi^2 ^and includes more than 119,654 residents [38,39].

### Data Collection

The BVFEP used ground-truthed methods in a two-stage approach to determine the access to fast food for residents living in the six rural counties [26,40]. In the first stage, trained observers systematically drove all highways (Interstate, U.S., and State), farm-to-market roads, and city or town streets/roads within the study area in order to identify all retail food venues. All traditional (supercenters, supermarkets, and grocery stores), convenience (convenience stores and food marts), and non-traditional (dollar stores, mass merchandisers, and pharmacies) food stores, full-service restaurants, and fast-food restaurants were enumerated, This process involved direct observation and on-site determination of geographic coordinates using a Bluetooth Wide Area Augmentation System (WAAS)-enabled portable GPS receiver and the World Geodetic System 1984 datum [27,40]. Table 1 provides a definition used to classify specific types of traditional and non-traditional food stores. Traditional fast-food restaurants were defined as limited-service restaurants where customers pay before eating. These outlets sell quick service foods that are ready for immediate consumption on premises, off premises, or foods that are delivered to the customer's location. Traditional fast-food restaurants did not include snack and nonalcoholic beverage bars [41,42]. In the second stage, an observational survey instrument was developed, tested, and administered in all fast-food restaurants and food stores by trained observers to determine the availability and variety of fast-food entrées and side dishes [26]. The enumerated census of food stores included 23 grocery stores/supermarkets and 143 convenience stores, which were considered to be non-traditional fast-food outlets [26]. Twelve (52.2%) grocery stores/supermarkets and 109 (76.2%) convenience stores marketed fast-food entrées.

**Table 1:** Definition of types of food stores used in this study [52]

***Supercenters or superstores***	Very large stores that primarily engage in retailing a general line of groceries in combination with general lines of new merchandise, such as apparel, furniture, and appliances (e.g., Super Wal-Mart, Super Kmart).
***Supermarkets***	Primarily engage in retailing a general line of food, supermarkets are larger in size (>20,000 sq ft), number of employees, and sales volume {Alwitt, 1997 #3550}. Chain store identification and number of parking spaces (>100) were used to distinguish supermarkets from grocery stores {Hale, 2004 #3377;Sharkey, 2009 #3556}.
***Grocery stores***	Primarily engage in retailing a general line of food, grocery stores are smaller in size, not identified as a chain store and have fewer than 100 parking spaces.
***Convenience stores or food marts***	Primarily engage in retailing a limited line of goods that generally includes milk, bread, soda, and snacks. The convenience store category also included convenience stores with gasoline and gasoline stations with convenience stores.
***Mass merchandisers***	Large, general merchandise "value" stores, such as Kmart, Target, and Wal-Mart.
***Dollar stores***	Limited-price general merchandise "value" stores, such as Dollar General or Family Dollar {Hale, 2004 #3377;Leibtag, 2005 #3375}.
***Pharmacies and drug stores***	Pharmacies and drug stores that were part of national chains (e.g., CVS, Walgreens).

Reprinted with permission: Sharkey et al. *International Journal of Health Geographics *2010, **9**:26 [52]

BVHA participants were recruited by a professional survey research firm using random digit dialed telephone screening and provided consent to participate in the survey. Details on the recruitment methodology have been previously published [43-45]. Briefly, prospective participants were initially contacted by telephone; a survey booklet, cover letter, small monetary incentive, and postage paid envelope were mailed to those adults who agreed to participate. Complete data on demographic characteristics and fast-food consumption were available for 1,409 adults who resided in one of the six rural counties (216 rural respondents were excluded because of missing data in the variables of interest). All participants were geocoded to their residence using Centrus Geocoder for ESRI ArcGIS™ with Tele Atlas street reference data. Street address includes streets, County Road, Private Road, or Farm-to-Market Road. Seventeen rural survey participants were unable to be geocoded and were not included in this sample.

Personal characteristics included age, gender, race/ethnicity, household income, household size (number of people living in household), employment status, and body mass index (BMI). Household income was categorized as poverty (≤100% Federal Poverty Level [FPL]), low income (101% to 199% FPL), and above low income (≥200% FPL). Body mass index (BMI) was calculated from self-reported height and weight, and was categorized as normal (<25 kg/m^2^), overweight (25-29.9 kg/m^2^), and obese (≥30 kg/m^2^). Employment status was dichotomized as employed full-time for wages vs. all others. The frequency of fast-food meal consumption was assessed with the following survey question: "How many times a week do you eat fast-food meals?" Six response categories ranged from "0" to "more than 6" times a week. This variable was modeled as a continuous variable given no dietary recommendation for fast-food consumption [46]. This question is similar to one that has been used in prior studies [10,13,17,18,46-51].

### Spatial access to fast-food

The network distance measure was computed for the distance along the road network; network distance was calculated with ESRI's Network Analysis extension in ArcInfo 9.2 from each BVHA participant's residence to the geographic position in front of each traditional fast-food restaurant and non-traditional fast-food outlet (convenience stores, supermarkets, and grocery stores that marketed fast-food items) [40]. Two criteria of potential spatial access to fast-food were calculated from the residence of each BVHA respondent: 1) proximity and 2) coverage. *Proximity *(minimum network distance) was used to measure distance to the nearest traditional fast-food restaurant, non-traditional fast-food outlet, or fast-food opportunity (traditional fast-food restaurant or non-traditional fast-food outlet). Separate network distances in miles were calculated from the residence of each participant to the nearest traditional fast-food restaurant or non-traditional fast-food outlet within the six-county study area [52]. Network data were provided by the 2003 Tele Atlas Dynamap Transportation version 5.2. *Coverage *(number of different shopping opportunities) was used to identify the number of different fast-food locations within a specified network distance of the respondent's residence [52]. While proximity is typically used to measure distance to the nearest food venue, coverage adds the dimension of variety and competition within a specific distance, regardless of administratively-defined areas [52]. Using the shortest network distance from each residence, Network Analyst computed the total number of traditional fast-food restaurants, non-traditional fast-food outlets, and all fast-food opportunities within 1, 3, and 5 mile network buffers to be consistent with previous work [52].

### Statistical Analysis

All statistical analyses were conducted with Release 11 of Stata Statistical Software (College Station, TX); *p *< 0.05 was considered statistically significant. Descriptive statistics were calculated for personal characteristics, spatial access to fast food (proximity and coverage), and frequency of fast-food meal consumption. Proximity and coverage measures of traditional fast-food restaurants was compared with non-traditional fast-food outlets, and all fast-food opportunities (fast-food restaurants, convenience stores, supermarkets, and grocery stores) by testing for equalities in mean, median, and distribution of measures using the Wilcoxon signed-ranks test [53]. Bivariate correlations between frequency of fast-food meal consumption and personal characteristics were estimated using Pearson's product-moment correlation. Three multiple variable regression models were fitted to determine the association of fast-food proximity (to nearest traditional fast-food restaurant, non-traditional fast-food outlet, and any fast-food opportunity) with frequency of fast-food meal consumption. In one model, spatial access was entered as the distance to the nearest traditional fast-food restaurant; in the second model, distance to the nearest non-traditional fast-food outlet; and in the third model, spatial access was defined as distance to the nearest fast-food opportunity, regardless of type. In all models, backward elimination was used for variables not significant (*p *< 0.10), which included race/ethnicity, household size, and employment. Since preliminary analysis showed a significant association of gender, an interaction variable was constructed for gender and spatial access (proximity and coverage). Nine multiple variable regression models were fitted to determine the association of fast-food coverage with frequency of fast-food meal consumption. In three models, spatial access was entered as the number of traditional fast-food restaurants within 1, 3, and 5 miles; in three models, spatial access was entered as the number of non-traditional fast-food outlets within 1, 3, and 5 miles; and in three models, spatial access was defined as the number of all fast-food opportunities within 1, 3, and 5 miles.

## Results

Personal characteristics, spatial access to fast food, and frequency of fast-food meals are shown in Table 2. Almost three-fourths of respondents were women and more than 34% were obese based on self-reported height and weight. Distance to the nearest non-traditional fast-food outlet was closer than to the nearest traditional fast-food restaurant; and the number of different venues for the purchase of fast food was greater for all fast-food opportunities than for traditional fast-food restaurants alone. The distances (proximity) and number (coverage) of traditional fast-food restaurants, non-traditional fast-food outlets, and all fast-food opportunities from each respondent's residence were different (Table 3). The distance to the nearest traditional fast-food restaurant was greater than to the nearest non-traditional fast-food outlet or to any fast-food opportunity. Within 1 and 5 miles of their residence, the number of different non-traditional fast-food outlets was greater than for traditional fast-food restaurants.

**Table 2:** Demographic characteristics, spatial access to fast food, and frequency of fast-food meals among rural adults (*n *= 1,409)

	**Mean ± SD (Median)**	**% (*n)***
***Demographic characteristics***		
Age, y	55.4 ± 15.2 (56)	
Women		73 (1030)
Race/ethnic minority^1^		17.6 (245)
Income		
Poverty (≤100% FPL)		18.0 (254)
Low income (101%-199% FPL)		14.7 (207)
BMI (kg/m^2^)		
Normal (<25)		31.6 (445)
Overweight (25-29.9)		34.3 (483)
Obese (≥30)		34.1 (481)
Household size^2^	2.5 ± 1.3 (2)	
Employed^3^		40.6 (559)
***Spatial access to fast food***		
Access to fast food (proximity), mi		
Nearest traditional fast-food restaurant	5.8 ± 5.4 (4.1)	
Nearest non-traditional fast-food outlet	3.2 ± 3.5 (1.6)	
Nearest fast-food opportunity^4^	3.0 ± 3.4 (1.5)	
Access to fast-food (coverage)^5^		
≤1 mi of the respondent's residence		
Traditional fast-food restaurants	1.7 ± 3.2 (0)	
Non-traditional fast-food outlets	1.9 ± 2.5 (0)	
All fast-food opportunities	3.6 ± 5.5 (0)	
≤3 mi of the respondent's residence		
Traditional fast-food restaurants	4.7 ± 7.4 (0)	
Non-traditional fast-food outlets	4.6 ± 6.4 (2)	
All fast-food opportunities	9.3 ± 13.7 (2)	
≤5 mi of the respondent's residence		
Traditional fast-food restaurants	5.8 ± 8.1 (2)	
Non-traditional fast-food outlets	6.0 ± 7.4 (4)	
All fast-food opportunities	11.8 ± 15.4 (6)	
***Fast-food meal consumption***		
Frequency of fast-food meals (per wk.)	1.3 ± 1.1 (1)	

FPL = Federal Poverty Level; BMI = body mass index

^1 ^Data on race/ethnic group membership provided by 1,402 respondents

^2 ^Household size = number of adults and children living in the household. Household size data provided by 1,397 respondents

^3 ^Employed = employed full-time for wages. Employment data provided by 1,384 respondents

^4 ^Distance to nearest traditional fast-food restaurant or non-traditional fast-food outlet, whichever is closest

^5 ^All fast-food opportunities = total of traditional fast-food restaurants and non-traditional fast-food outlets

**Table 3:** Comparison of proximity and coverage measures of spatial access between traditional fast-food restaurants, non-traditional fast-food outlets, and all fast-food opportunities, using the Wilcoxon Signed-Ranks Test (*n *= 1,409)

	*Z*-statistic	*P*-value
*Proximity*		
Traditional fast-food restaurant vs. non-traditional fast-food outlet	22.99	<0.0001
Traditional fast-food restaurant vs. all fast-food opportunities	30.51	<0.0001
Non-traditional fast-food outlet vs. all fast-food opportunities	14.10	<0.0001
*Coverage (*≤ *1 mile)*		
Traditional fast-food restaurant vs. non-traditional fast-food outlet	-12.50	<0.0001
Traditional fast-food restaurant vs. all fast-food opportunities	-25.40	<0.0001
Non-traditional fast-food outlet vs. all fast-food opportunities	-22.49	<0.0001
*Coverage (*≤ *3 miles)*		
Traditional fast-food restaurant vs. non-traditional fast-food outlet	-1.67	0.094
Traditional fast-food restaurant vs. all fast-food opportunities	-29.24	<0.0001
Non-traditional fast-food outlet vs. all fast-food opportunities	-25.31	<0.0001
*Coverage (*≤ *5 miles)*		
Traditional fast-food restaurant vs. non-traditional fast-food outlet	-5.64	<0.0001
Traditional fast-food restaurant vs. all fast-food opportunities	-31.39	<0.0001
Non-traditional fast-food outlet vs. all fast-food opportunities	-27.64	<0.0001

In the all-adjusted regression models for proximity (Table 4), increased age, poverty-level income, and being female were all associated with less-frequent weekly consumption of fast-food meals. Conversely, being overweight or obese was associated with increased fast-food meal consumption. The greater the distance to the nearest traditional fast-food restaurant, non-traditional fast-food outlet, or all fast-food opportunities was associated with less frequent consumption of fast-food meals. The effect of the interaction of gender and spatial access show that the effect of distance to the nearest traditional fast-food restaurant, non-traditional fast-food outlet, and any fast-food opportunity is greater in magnitude for women than for men; in other words, greater distance was associated with more frequent consumption of fast-food meals by women than men. This was opposite of the main effect of gender or proximity.

**Table 4:** Association of personal characteristics and proximity to nearest traditional fast-food restaurant, non-traditional fast-food outlet, and all fast-food opportunities with frequency of weekly consumption of fast-food meals in 1,409 rural adults, using multiple variable regression

	Regression coefficient		
***Personal characteristics***			
Age, y	-0.018***	-0.018***	-0.018***
Household income^1^			
Poverty (≤100% FPL)	-0.264***	-0.258***	-0.262***
Low income (101%-199% FPL)	-0.150	-0.143	-0.146
BMI (kg/m^2^)^2^			
Overweight (25-29.9)	0.144*	0.142*	0.142
Obese (≥30)	0.267***	0.275***	0.276***
Gender^3^			
Women	-0.477***	-0.334**	-0.357**
***Spatial access to fast food***			
Traditional fast-food restaurant	-0.045***		
Non-traditional fast-food outlet		-0.044**	
All fast-food opportunities			-0.048**
***Interaction (gender and spatial access)***			
Traditional fast-food restaurant	0.047***		
Non-traditional fast-food outlet		0.039*	
Fast-food opportunity			0.048**

Intercept	2.72	2.60	2.60
*R*^*2 *^of model	0.102	0.094	0.094

FPL = Federal Poverty Level

^1 ^Reference category for household income: Above low income (≥200% FPL)

^2 ^Referent category for BMI (body mass index): Normal (<25 kg/m^2^)

^3 ^Referent category for gender: men

Level of statistical significance at level of * *p *< 0.05, ** *p *< 0.01, *** *p *< 0.001

In the models for coverage (Tables 5, 6, and 7), age, poverty-level income, and obesity remained significant correlates of fast-food consumption; however, the main effect of gender was no longer significant. The main effect of coverage within 1, 3, and 5 miles of residences were significant for an increased number of traditional fast-food restaurants, non-traditional fast-food outlets, or all fast-food opportunities. The effect size was greater for coverage within 1 mile than within 3 or 5 miles. The effect of the interaction of gender and coverage is greater for women than for men; a greater variety of different traditional fast-food restaurants, non-traditional fast-food outlets, or all fast-food opportunities were associated with less frequent consumption of fast-food meals by women than men. The effect size was greater for coverage within 1 mile than within 3 or 5 miles of the residence.

**Table 5:** Association of personal characteristics and one-mile coverage of fast-food restaurant, non-traditional fast-food outlet, and all fast-food opportunities with frequency of weekly consumption of fast-food meals in 1,409 rural adults, using multiple variable regression

	Regression coefficient		
	Model 1	Model 2	Model 3
***Personal characteristics***			
Age, y	-0.019***	-0.019***	-0.019***
Household income^1^			
Poverty (≤100% FPL)	-0.266***	-0.261***	-0.262***
Low income (101% to 199% FPL)	-0.153	-0.153	-0.153
BMI (kg/m^2^)^2^			
Overweight (25-29.9)	0.146*	0.142	0.145*
Obese (≥30)	0.274***	0.270***	0.272***
Gender^3^			
Women	-0.080	-0.087	-0.072
***One-mile coverage of fast food***			
Traditional fast-food restaurant	0.057**		
Non-traditional fast-food outlet		0.059**	
All fast-food opportunities			0.031**
***Interaction (gender and one-mile coverage)***			
Traditional fast-food restaurant	-0.065**		
Non-traditional fast-food outlet		-0.056*	
Fast-food opportunity			-0.033**

Intercept	2.35	2.34	2.33
*R*^*2 *^of model	0.095	0.094	0.095

FPL = Federal Poverty Level

^1 ^Reference category for household income: Above low income (≥200% FPL)

^2 ^Referent category for BMI (body mass index): Normal (<25 kg/m^2^)

^3 ^Referent category for gender: men

Level of statistical significance at level of * *p *< 0.05, ** *p *< 0.01, *** *p *< 0.001

**Table 6:** Association of personal characteristics and three-mile coverage of fast-food restaurant, non-traditional fast-food outlet, and all fast-food opportunities with frequency of weekly consumption of fast-food meals in 1,409 rural adults, using multiple variable regression

	Regression coefficient		
	Model 1	Model 2	Model 3
***Personal characteristics***			
Age, y	-0.019***	-0.019***	-0.019***
Household income^1^			
Poverty (≤100% FPL)	-0.260***	-0.259***	-0.259***
Low income (101% to 199% FPL)	-0.149	-0.149	-0.149
BMI (kg/m^2^)^2^			
Overweight (25-29.9)	0.140	0.140	0.140
Obese (≥30)	0.267***	0.268***	0.267***
Gender^3^			
Women	-0.100	-0.107	-0.102
***Three-mile coverage of fast food***			
Traditional fast-food restaurant	0.020**		
Non-traditional fast-food outlet		0.020*	
All fast-food opportunities			0.010**
***Interaction (gender and three-mile coverage)***			
Traditional fast-food restaurant	-0.020*		
Non-traditional fast-food outlet		-0.020	
Fast-food opportunity			-0.010*

Intercept	2.36	2.36	2.36
*R*^*2 *^of model	0.094	0.092	0.093

FPL = Federal Poverty Level

^1 ^Reference category for household income: Above low income (≥200% FPL)

^2 ^Referent category for BMI (body mass index): Normal (<25 kg/m^2^)

^3 ^Referent category for gender: men

Level of statistical significance at level of * *p *< 0.05, ** *p *< 0.01, *** *p *< 0.001

**Table 7:** Association of personal characteristics and five-mile coverage of fast-food restaurant, non-traditional fast-food outlet, and all fast-food opportunities with frequency of weekly consumption of fast-food meals in 1,409 rural adults, using multiple variable regression

	Regression coefficient		
	Model 1	Model 2	Model 3
***Personal characteristics***			
Age, y	-0.019***	-0.019***	-0.019***
Household income^1^			
Poverty (≤100% FPL)	-0.260***	-0.261***	-0.260***
Low income (101% to 199% FPL)	-0.147	-0.145	-0.146
BMI (kg/m^2^)^2^			
Overweight (25-29.9)	0.138	0.139	0.139
Obese (≥30)	0.266***	0.266***	0.266***
Gender^3^			
Women	-0.062	-0.050	-0.055
***Five-mile coverage of fast food***			
Traditional fast-food restaurant	0.023***		
Non-traditional fast-food outlet		0.024**	
All fast-food opportunities			0.012**
***Interaction (gender and five-mile coverage)***			
Traditional fast-food restaurant	-0.024**		
Non-traditional fast-food outlet		-0.025**	
Fast-food opportunity			-0.013**

Intercept	2.32	2.32	2.32
*R*^*2 *^of model	0.096	0.095	0.095

FPL = Federal Poverty Level

^1 ^Reference category for household income: Above low income (≥200% FPL)

^2 ^Referent category for BMI (body mass index): Normal (<25 kg/m^2^)

^3 ^Referent category for gender: men

Level of statistical significance at level of * *p *< 0.05, ** *p *< 0.01, *** *p *< 0.001

## Discussion

Although research findings indicate that fast-food restaurants dominate the food landscape and the demand for fast food is increasing, there are few studies that have examined the association of spatial access to fast food on the frequency of consuming fast-food meals, especially among rural adults [19,36]. This is critical considering relationships reported between frequency of fast-food meal consumption and increased caloric and fat intake and reduced intake of key nutrients [1,10,14,18,48,49,54,55]. However, studies of fast-food consumption rarely have considered measured access to fast food as a contributing factor [1,6,10,14,17,18,33,49,56]. Findings from this study extend the understanding of the association of personal characteristics and access to fast food on frequency of fast-food meal consumption among adults who reside in rural areas. This is apparently the first study, to our knowledge, that: 1) uses ground-truthed data on access and availability of fast food from traditional and non-traditional fast-food outlets, such as convenience stores, supermarkets, and grocery stores and 2) provides a comprehensive picture of the fast-food environment for a large rural area. Our analyses revealed that both dimensions of spatial access - proximity and coverage - indicated significantly better access to fast food from non-traditional outlets than from traditional fast-food restaurants alone. A second important finding was that spatial access to fast food, in terms of proximity and coverage, was associated with frequency of fast-food meals especially among women. Several findings require further discussion.

### Fast-food consumption

Unlike studies that asked specifically about fast-food restaurant use from major national/international chains, our rural respondents were asked about weekly, general fast-food meal consumption [10,13,14,17-19,34,47,49-51,56]. By not restricting the responses to major chains, respondents were able to consider the more prevalent local and regional fast-food restaurants as well as other fast-food venues found in rural areas. On the average 1.3 fast-food meals were consumed by the 1,409 rural adults in our sample. However, compared with other studies, the rural men and women in this sample consumed fewer fast-food meals than reported in predominately urban areas [17,46].

### Potential spatial access to fast-food

Compared with a previous study of rural areas in three states that measured access by self-reported walking time from residence to the nearest fast-food restaurant [19], this study used ground-truthed methods to identify, geocode, and calculate the network distance to the nearest traditional and non-traditional fast-food venues and the number of fast-food venues within a specified distance of the residence. Other studies have used perceived access [7,57], secondary lists [11,34,35], or a combination of direct observation and telephone books [36]. Prior work has shown that the use of secondary lists distorts representations of the food environment, and that ground-truthing is the most accurate measure [27,40], especially in rural areas [27,40]. Considering that the average distance to the nearest traditional fast-food restaurant was 5.8 miles and 3.2 miles to the nearest non-traditional fast-food outlet, walking time was an inappropriate measure in our rural area. Spatial access to fast food, using both proximity (distance to the nearest location) and coverage (number of fast-food locations) measures, was significantly better to non-traditional fast-food outlets than to traditional fast-food restaurants alone. On average, the distance to the nearest fast-food opportunity was nearly half the distance to the nearest fast-food restaurant. There was twice the number of all fast-food opportunities compared to traditional fast-food restaurants within all three buffer distances. This suggests that limiting measures of spatial access to traditional fast-food restaurants alone or to major chains may significantly underestimate "true" exposure to fast food [24]. Our work overcomes limitations of prior studies that examined fast-food consumption, but failed to consider spatial access as a determinant [10,46,47].

Residential distance was associated with fast-food consumption. In regression models, after adjustment for personal characteristics, increased distance to the nearest traditional fast-food restaurant, non-traditional fast-food outlet, or all fast-food opportunities, was associated with decreased weekly frequency of fast-food meals. This is in contrast to the work of Jeffery and colleagues in an urban area where they found that proximity to the home was not related to frequency of fast-food consumption [11]. In addition, coverage for traditional fast-food restaurants, non-traditional fast-food outlets, and all fast-food opportunities was associated with fast-food consumption, regardless of the coverage area. However, the magnitude of the association of coverage, or the number of the different venues, for traditional fast-food restaurants, non-traditional fast-food outlets, or all fast-food opportunities was almost three times greater with one-mile of the residence, compared with three- and five-mile buffer areas. This suggests that both proximity to the nearest fast-food venue and the number of different choices were associated with fast-food consumption. Further, proximity and coverage for non-traditional fast-food outlets had a similar independent association with fast-food consumption as traditional fast-food restaurants. It is possible that rural residents may be similarly attracted to traditional and non-traditional fast-food venues.

The interaction of gender and spatial access was associated with fast-food consumption, controlling for the independent association of gender and spatial access. The association of distance and coverage on fast-food consumption was greater for women than for men. For comparable increase in distance to the nearest traditional fast-food restaurant, non-traditional fast-food outlet, or any fast-food opportunity, the association for women was for a decrease in frequency of fast-food meals, compared with men. Similar significant associations were observed for coverage; the greater the coverage, the less frequent consumption of fast-food meals for women. The interaction revealed that proximity and coverage do not have the same direction of association as the independent effect of proximity and coverage. There may be gender-based inequities in access to transportation, and given the average distances to both traditional fast-food restaurants and non-traditional fast-food outlets, women without available transportation may visit fast-food destinations less often. The coefficients for interaction with traditional fast-food restaurants and non-traditional fast-food outlets were similar in magnitude, indicating an association on frequency of fast-food meal consumption among women. This finding may be associated with men and women having different travel activity patterns that are influenced by family and work demands, which also influence interactions within the environment [58-62]. The relationships between better access to fast food based on home residence and less frequent fast-food consumption for women may be related to less-frequent use of fast-food restaurants than men [54], and daily travel patterns. One possible explanation is that women may be less influenced by the food outlets nearest their home because of family, social, volunteer/professional responsibilities that require travel beyond a home-centered activity space; women's travel patterns may be more varied from accommodating to changing work schedules or family demands compared to men's, whose travel patterns between work and home are relatively more stable. Another possibility may be that women, who were less likely to be employed for wages than men, consumed less fast food because they spent more time at home or other locations where fast food was not readily available. Alternatively, women may have distinct motivations for seeking out fast food, which may explain these findings [6]. Understanding men and women's interactions with the food environment in context with other behaviors and dimensions of the environment, such as the location and distribution of nonfood destinations (e.g., work, school, childcare facilities, banking and other service centers, and retail outlets), may elucidate the distinct relationship between access to fast food and consumption in men compared with women.

It is especially important to note that, in addition to spatial access to fast food, personal characteristics were significantly associated with frequent fast-food consumption. Overall, poverty status was associated with fewer fast-food meals. This is opposite of a previous finding that lower income was associated with more frequent fast-food use [56], and similar to one where higher income was associated with more frequent fast-food consumption [54].

Obesity was consistently associated with increased fast-food consumption. Prior studies have shown mixed results for the relationship between obesity and fast-food consumption [10,11,36]. Interestingly, three additional personal characteristics were not independently associated with fast-food consumption in our rural sample; namely, race/ethnicity, household size, and employment status. Correlations between these three characteristics and household income may explain the lack of statistical significance in the regression models.

### Strengths

There are several methodological strengths to this study. First, data for the location and availability of fast food in this large rural area were collected using ground-truthed methods, which have been shown to be more accurate in small-town and rural areas than secondary or publicly available lists [27,40,63]. Second, data were not restricted to fast-food restaurants or the major fast-food restaurant chains, but included all traditional fast-food restaurants and non-traditional fast-food outlets, such as convenience stores, supermarkets, and grocery stores that market fast-food meals. This provides a comprehensive picture of the fast-food environment. Third, our measures of access included two dimensions; namely, proximity (distance to the nearest location) and coverage (number of locations within a specified area). Finally, data on fast-food consumption was collected from a large sample of rural men and women who participated in a community health status assessment.

### Limitations

Although data permitted the examination of potential spatial access to fast food from the residence, travel patterns, car ownership, origin of food purchase trips, or distance to employment were not available in the data. It is not known whether other personal (e.g., fast-food preferences, available transportation) or environmental aspects, including the location and distribution of nonfood destinations (e.g., work, schools, banking and other service centers, retail outlets), may be more relevant than residential access and eating behavior measures for understanding fast-food consumption in rural women. Future work with rural families will allow documentation of other missing information, such as specific fast foods and amount consumed, location of fast-food purchase, and criteria for selecting fast-food outlet. As with other reports of food consumption, whether fast food or not, the assumption is that data represent "usual" intake, which may not be the case. A further limitation is the lack of definition of fast food in the survey and lack of validity that may result from a difference in perceived meaning of the term "fast food" among respondents. Finally, our rural study area may not be representative of other rural areas.

## Conclusion

Despite these limitations, this study furthers our knowledge about potential spatial access to fast food and the frequency of fast-food meal consumption among rural adults. Results from this study provide impetus for identifying and understanding the complex relationship between access to all fast-food opportunities, rather than to traditional fast-food restaurants alone, and fast-food consumption. The results also indicate the importance of further examining the complex role of gender in fast-food consumption. Furthermore, this study emphasizes the need for health promotion and policy efforts to consider all sources of fast food as part of promoting healthful food choices with rural adults.

## Competing interests

The authors declare that they have no competing interests.

## Authors' contributions

JRS developed the original idea for the study. JRS conducted the analysis and wrote the first draft of the paper. SAH conducted all geocoding and mapping. JRS, CMJ, SAH, and WRD read and approved the final manuscript.
